# Editorial: Application of biotechnology on the high-value development of marine bioresource

**DOI:** 10.3389/fbioe.2022.1037283

**Published:** 2022-10-28

**Authors:** Zedong Jiang, Haijin Mou, Yuya Kumagai, Hideki Kishimura

**Affiliations:** ^1^ College of Ocean Food and Biological Engineering, Jimei University, Xiamen, Fujian, China; ^2^ College of Food Science and Engineering, Ocean University of China, Qingdao, Shandong, China; ^3^ Faculty of Fisheries Sciences, Hokkaido University, Hakodate, Hokkaido, Japan

**Keywords:** biotechnological application, marine macromolecule, astaxanthin, cutinase, volatile fingerprint

The exploration and high-value utilization of marine bioresources is an important hotspot in numerous research and application fields, including global marine biology, food science, medical science, and others. Modern biotechnology, including genetic engineering, enzyme engineering, and microbial engineering, plays a critical role in the high-value development and utilization of marine bioresources and is receiving increasing attention. As we know, the exploration of new genes, new enzymes, and special environmental microbial material sources from the ocean are inseparable from the means of biotechnology. Moreover, the effective extraction, processing, and transformation of functional components of marine organisms, such as polysaccharides, peptides, lipids, and others, can also be achieved through biotechnology. Recently, the application of biotechnology, such as bioprocessing technology, biological preservation technology, food fermentation technology, etc., in improving the nutrition, flavor, and quality of marine foods as well as extending their shelf life, has aroused extensive research interest. In addition, emerging technologies applied in the fields of marine food safety and quality control, such as chip technologies, serological reaction technology, immunological reaction technology, etc., also require the support of biotechnology and have gradually become one of the focuses. Therefore, advanced marine biological technology is the basis and key to the high-value development and utilization of marine bioresources.

This Research Topic includes 6 original research articles, which mainly focuses on the preparation and functional evaluation of marine macromolecular functional components (including polysaccharide, proteins, and peptides), the biological preparation of marine small molecular functional components, the exploration of marine new enzyme helping to eliminate environmental plastic pollutants, and flavor identification and analysis for fermented fish.

Two articles focus on the preparation and functional evaluation of polysaccharides from seaweed *Laminaria japonica* and bioactive peptides from large hybrid sturgeon by directed enzymatic hydrolysis. One article concerned the medical therapeutic properties and application of collagen and collagen peptides from jellyfish. The potential action mechanisms of these macromolecules have been further elucidated. Fu et al. described a novel method of preparing low-molecular-weight polysaccharides from *L. japonica* by high-pressure pretreatment combined with enzymatic degradation and further evaluated their anti-obesity functions in high-fat-diet (HFD)-fed mice model. Numerous polysaccharides derived from seaweeds have been found to ameliorate HFD-induced metabolic syndromes and obesity, which have attracted extensive attention for their possible mechanisms. This work demonstrates that these polysaccharides have significant effects on obesity by improving the intestinal microbiota and increasing the synthesis of SCFAs with the *in vitro* degradation system and HFD-fed mice model. Marine fish-derived bioactive peptides have potential health benefits, of which the high-value utilization has become a trend in food, medical, and cosmetic industries. Chen et al. optimized the hydrolysis conditions of sturgeon skin collagen hydrolysate (SSCH) using DPPH radical scavenging activity as an indicator based on the Box-Behnken design of response surface methodology. Under optimal hydrolysis conditions, the obtained SSCH exhibited potent antioxidant activity. Among the SSCH, low-molecular-weight SSCH fraction (SSCH-L) significantly alleviates the oxidative damage caused by UVB irradiation in both *in vitro* (mouse fibroblast L929 cells) and *in vivo* (zebrafish) models. More interestingly, SSCH-L exhibited the potential to repair UVB-irradiated zebrafish skin damage. This anti-photodamage effect might be mainly involved in down-regulated pro-inflammatory cytokines and the expression level of matrix metalloprotease by suppressing the JNK MAPK signaling pathway. Based on the 3D-QSAR modeling prediction, the peptide DPFRHY highly matched to SSCH-L was screened, which exhibited significant activity in repairing UVB radiation damage and has potential development as natural anti-photodamage materials. Felim et al. compared the efficacy of collagen and collagen peptides from jellyfish (*Rhopilema esculentum*), other marine collagen, and glycine in the treatment of osteoarthritis by using a surgery-induced osteoarthritis rat model. This work found that collagen and glycine have protective effects on cartilage degradation in osteoarthritis by daily oral administration for 6 weeks, whereas collagen peptides showed promotion properties of cartilage regeneration. Especially, collagen peptide at a dose of 5 mg/kg body weight showed the most significant protecting and regenerating effects on the cartilage in the knee, having the best potential to treat osteoarthritis.

Astaxanthin, belonging to marine small molecular functional components, is an important marine bioresource with antioxidant, anti-inflammatory, anti-aging, and anti-tumor properties. *P. rhodozyma* belongs to basidiomycetes and is one of the major biological sources of astaxanthin. Metabolic regulation is an effective method for improving astaxanthin production in *P. rhodozyma*. Li et al. focused on systematically screening single regulators could help to analyze their regulatory functions and effects on metabolic pathways. What’s more, astaxanthin’s biosynthetic and competing pathways could be controlled to the best effect for improving the astaxanthin in *P. rhodozyma*.

Enzyme engineering is an essential means to discover a novel enzyme and further improve or customize its properties. Liu et al. described the biochemical characteristics of a novel cutinase (MtCut) with polyethylene terephthalate (PET) hydrolyzing activity from a deep-sea bacterium *Marinactinospora thermotolerans* DSM45154. PET is a synthetic aromatic polyester used to produce textile fibers and resins for single-use packaging and beverage bottles. MtCut efficiently hydrolyzed PET at ambient temperatures without significant inhibition by its hydrolysates. This work further clarified key regions closely associated with enzyme thermostability that will provide invaluable insights into the catalysis and thermostability of cutinase-like PET hydrolases.

Fermentation can help to improve the sensory characteristics, nutritional properties, texture, and microbial safety of foods. The flavor is an important sensory characteristic of fermented foods. One article established a volatile fingerprint of fermented mandarin fish using electronic nose and GC–IMS analyses, which allowed to clearly distinguish different stages during the fermentation of mandarin fish. This work provided technical support for flavor real-time monitoring and quality control of fish fermentation.

Although the research results collected on this Research Topic will provide scientific basis and technical support for the high-value development and utilization of marine bioresources, there are not exhaustive. Gene engineering, protein engineering, etc., which are the key application technologies in modern biotechnology, not only effectively improve the quality of high-value development and utilization of marine bioresources, but also effectively improve their productivity. Reasonable application of biotechnology can successfully improve the property of a traditional marine organism or transform it into new specie, thus enriching the available marine bioresources. Also, various marine ecological environments contain abundant bioresources that produce special bioactive substances, such as functional proteins, polysaccharides, unsaturated fatty acids, pigments, terpenes, and others. They are important raw materials’ resources of medicine, food, and chemical, which needs to be discovered and excavated.

